# The Value of Multimodal Ultrasound Based on Machine Learning Algorithms in the Diagnosis of Benign and Malignant Thyroid Nodules of TI-RADS Category 4: A Single-Center Retrospective Study

**DOI:** 10.2174/0115734056404285251006113649

**Published:** 2025-10-15

**Authors:** Minglei Ren, Zengdi Yang, Ying Fu, Zhichun Chen, Ying Shi, Yongyan Lv

**Affiliations:** 1 Department of Ultrasound, The 901st Hospital of the Joint Logistics Support Force of PLA, Hefei, 230031, China; 2 Department of Ultrasound, Peking University Third Hospital, No. 49 North Garden Road, Haidian District, Beijing, 100191, China; 3 Center of Minimally Invasive Interventional Therapy, Dongfang Hospital Affiliated to Tongji University, No. 150, Jimo Road, Pudong District, Shanghai, 200120, China

**Keywords:** Thyroid nodule, Machine learning algorithm, Contrast-enhanced ultrasound, Shear wave elastography, Retrospective study, Ultrasound characteristics

## Abstract

**Introduction:**

Ultrasound is routinely used for thyroid nodule diagnosis, yet distinguishing benign from malignant TI-RADS category 4 nodules remains challenging. This study has integrated two-dimensional ultrasound, shear wave elastography (SWE), and contrast-enhanced ultrasound (CEUS) features via machine learning to improve diagnostic accuracy for these nodules.

**Methods:**

A total of 117 TI-RADS 4 thyroid nodules from 108 patients were included and classified as benign or malignant based on pathological results. Two-dimensional ultrasound, CEUS, and SWE were compared. Predictive features were selected using LASSO regression. Feature importance was further validated using Random Forest, SVM, and XGBoost algorithms. A logistic regression model was constructed and visualized as a nomogram. Model performance was assessed using receiver operating characteristic (ROC) analysis, calibration curves, and decision curve analysis (DCA).

**Results:**

Malignant nodules exhibited significantly elevated serum FT3, FT4, FT3/FT4, TSH, and TI-RADS scores compared to benign lesions. Key imaging discriminators included unclear boundaries, aspect ratio ≥1, low internal echo, microcalcifications on ultrasound; enhancement degree, circumferential enhancement, and excretion on CEUS; and elevated SWE values (Emax, Emean, Esd, etc.) and altered CEUS quantitative parameters (PE, WiR, WoR, etc.) (all *P*< 0.05). A nomogram integrating four optimal predictors, including Emax, FT4, TI-RADS, and ∆PE, demonstrated robust predictive performance upon validation by ROC, calibration, and DCA curve analysis.

**Discussion:**

The nomogram incorporating Emax, FT4, TI-RADS, and ∆PE showed high predictive accuracy, particularly for papillary carcinoma in TI-RADS 4 nodules. Its applicability may, however, be constrained by the single-center retrospective design and limited pathological coverage.

**Conclusion:**

The multimodal ultrasound-based machine learning model effectively predicted malignancy in TI-RADS category 4 thyroid nodules.

## 1 INTRODUCTION

The thyroid gland, which is composed of two lobes connected by a central isthmus, is the largest endocrine gland in the human body. In adults, it weighs approximately 20-30 grams. Epidemiological surveys indicate that the overall prevalence of thyroid diseases is between 4% and 7% [1]. Most patients have normal thyroid function and no obvious symptoms. The most common of these are thyroid nodules, which are abnormal proliferations of thyroid tissue that form masses capable of moving up and down with swallowing. Approximately 7-15% of these nodules may progress to malignancy and develop into thyroid cancer [2]. According to statistics, in 2020, there were about 586,000 new cases of thyroid cancer and about 44,000 deaths worldwide [3]. Early diagnosis of thyroid cancer is crucial for improving the prognosis of thyroid cancer. The assessment of thyroid nodules can be conducted using various imaging techniques, including ultrasound examination, computed tomography, and magnetic resonance imaging, among others. High-frequency ultrasound, due to its advantages of non-invasiveness, repeatability, cost-effectiveness, and convenience, has become the preferred screening method for clinical thyroid nodules, and it holds significant guiding importance in the qualitative diagnosis and subsequent treatment of thyroid nodules [4]. In order to standardize and reduce the diagnostic discrepancies of thyroid nodules, the American College of Radiology (ACR) proposed the Thyroid Imaging Reporting and Data System (ACR TI-RADS) [5] in 2017, which assigns different weighted scores based on the ultrasound characteristics of thyroid nodules and classifies them into categories 1-5 according to the total score. Among them, 4 types of lesions are defined as potentially malignant, with a malignancy rate of 3.6%-91.9% [6]. The characteristics of ultrasound diagnosis are distinct in category 3 and category 5 nodules, while the diagnostic features of category 4 thyroid nodules exhibit overlap. This indicates that nodules of different pathological types may present similarities in morphology, blood flow, metabolism, and symptomatology. Such overlap increases the complexity of diagnosis, necessitating that physicians consider multiple factors when assessing the nature of the nodules, including but not limited to the morphological characteristics of the nodules, blood flow conditions, metabolic activity, and the specific symptoms of the patient. Therefore, the diagnosis of these types of nodules is relatively challenging and urgent. With the continuous development of various examination technologies, different new ultrasound techniques are being increasingly integrated into the diagnosis of thyroid nodules. Contrast-enhanced ultrasound (CEUS) enhances the scattering signals of blood flow in the human body through the intravenous injection of ultrasound contrast agents, thereby allowing for real-time dynamic observation of the microvascular perfusion information of tissues, in order to improve the detection rate of lesions and differentiate between benign and malignant lesions [7]. The contrast agent used in CEUS examinations has minimal side effects and can be excreted through the pulmonary circulation. In clinical diagnostic work, it has demonstrated significant clinical reference value in areas, such as the liver and breast [8, 9], while also gradually playing a more important role in the diagnosis of thyroid nodules [10]. However, the assessment of the perfusion status of thyroid nodules through imaging is performed by ultrasound physicians, which carries a certain degree of subjectivity. Shear wave elastography (SWE), as a novel quantitative diagnostic method, can provide quantitative measurements of tissue stiffness, thereby reflecting the elastic characteristics of the interior and surrounding areas of the nodules, effectively enhancing the diagnostic performance of thyroid nodules [11]. However, some solid nodules may exhibit a “window” phenomenon, where the elasticity value inside the nodule is zero, while the elasticity value of the surrounding tissue outside the nodule is greater than zero. This phenomenon is considered a potential malignant sign; however, its presence does not necessarily indicate that the nodule is malignant [12]. Therefore, SWE should be combined with various imaging techniques for comprehensive assessment.

In the past decade, artificial intelligence has become a hot topic both within and outside the academic community, with machine learning being a subset of artificial intelligence. Its typical processes can be divided into designing algorithms for computers to learn automatically, training algorithm models with data to enable the algorithms to analyze patterns through data, and classifying and predicting unknown data based on the patterns learned by the algorithms [13]. With the research on various diseases in the field of medicine, including diagnosis, pathogenic factors, and treatment, a large amount of medical data can be trained using machine learning algorithms and widely applied in the field of ultrasound image recognition [14, 15]. Furthermore, significant achievements have been made in distinguishing between benign and malignant thyroid nodules [16, 17], differentiating clinical subtypes of thyroid nodules [18, 19], and assisting ultrasound specialists in improving diagnostic efficacy [20].

Based on this, this study combined the clinical indicators and multimodal ultrasound feature parameters of patients with TI-RADS category 4 thyroid nodules, including conventional two-dimensional ultrasound features, contrast-enhanced ultrasound features and parameters, as well as shear wave elastography parameters, to construct a predictive model for the malignancy of TI-RADS category 4 thyroid nodules using machine learning algorithms, thereby improving the accuracy of early detection, accurate diagnosis, and effective treatment of thyroid cancer.

## 2 MATERIALS AND METHODS

### 2.1 Patient Selection

A retrospective analysis of 1,980 patients with thyroid nodules who visited the 901 Hospital of the PLA Joint Logistic Support Force from January 2021 to December 2023 was conducted. After applying the inclusion and exclusion criteria, a total of 117 nodules from 108 patients were selected as the sample of this study.

Inclusion criteria were as follows: (1) malignant dangerous stratification based on ACR TI-RADS category 4 nodules, with clear surgical pathological results; (2) the patient's two-dimensional ultrasound, CEUS, and SWE examination data being complete; (3) age ≥ 18 years. Exclusion criteria were as follows: (1) patients who cannot tolerate contrast agents; (2) those who have undergone thyroid surgery, radiotherapy, radionuclide therapy, and other related treatments; (3) patients with other malignant tumors; (4) incomplete clinical data; (5) poor quality of ultrasound images. To ensure patient privacy, all patient information was de-identified prior to analysis. This study was based on existing clinical data, and the use of all data complied with the ethical principles outlined in the Declaration of Helsinki and its 2013 revision. The research plan and data collection were reviewed and approved by the ethics committee of this hospital (approval no.: 202503007), and all patients included in the study have signed informed consent forms. The flowchart of this study is shown in Fig. (**1**).

### 2.2 Two-dimensional Ultrasound Image Acquisition

An Aixplorer V ultrasound system (SuperSonic Imagine, Aix-en-Provence, France) equipped with linear array probes operating at frequencies of 2–10 MHz and 4–15 MHz was used. Ultrasound examinations and image acquisition were conducted by two physicians with over ten years of experience in thyroid ultrasound. In cases where the results were inconsistent, a senior physician discussed the findings collectively and made the final determination. The patient was positioned in a supine position, with the neck extended to adequately expose the thyroid region. Continuous scanning of multiple sections was performed, primarily recording the location of nodules, nodule size (maximum diameter), nodule composition, echogenicity, aspect ratio, margins, and strong echogenicity. Patients scoring between 4-6 according to the ACR TI-RADS scoring criteria [5] were classified as ACR TI-RADS category 4.

### 2.3 SWE

The largest cross-section of the thyroid nodule was first identified under two-dimensional ultrasound. Subsequently, the system was switched to shear wave elastography (SWE) mode, and the probe was gently placed on the neck, ensuring minimal pressure. Patients were instructed to hold their breath during image acquisition. A 2-mm sampling box was used to compare the stiffest region within the nodule to the corresponding area of normal thyroid tissue. Real-time SWE parameters, including maximum Young’s modulus (Emax), mean Young’s modulus (Emean), minimum Young’s modulus (Emin), and standard deviation (Esd), were obtained. Each measurement was repeated three times, and the average values were calculated. All SWE images were retained for analysis.

### 2.4 CEUS

After image acquisition of the patients’ SWE was completed, the system was switched to contrast mode. The SonoVue contrast agent was selected, and 5 mL of 0.9% sterile sodium chloride injection was added to the vial containing the lyophilized powder. The vial was then shaken until the powder was fully dispersed and dissolved. The microbubble suspension was drawn into the syringe and immediately injected into the peripheral vein. Each injection of Shenovi suspension was administered at a volume of 2.4 mL, followed by a flush with 5 mL of 0.9% sterile sodium chloride injection. The contrast agent perfusion of the patient's thyroid nodule was observed and recorded for 2 minutes, after which the images were exported in DICOM format. Off-line analysis was performed using Vue Box@ external perfusion software to obtain the temporal intensity curve and obtain relevant parameters, including PE (peak intensity), WiR (inflow ratio), WoR (outflow ratio), WiAUC (area under the wash-in phase curve), WoAUC (area under the wash-out phase curve), WiWoAUC (inflow and outflow phase AUC), WiPI (inflow phase perfusion index), and ΔPE and ΔWiR by calculating the difference between normal glandular tissue and nodule parameters, ΔWoR, ΔWiAUC, ΔWiWoAUC, ΔWoAUC, ΔWiPI, and other data.

### 2.5 Statistical Analysis

The data were analysed using R4.4.0 (R Foundation, Vienna, Austria) and Python (Python Software Foundation, Beaverton, Oregon, USA). The continuous data were tested by the Kolmogorov-Smirnov test, and the data conforming to the normal distribution have been expressed as x̅±s, and then the t-test was employed. Data that did not conform to the normal distribution have been expressed as M (Q1,Q3) using the Mann-whitey U test. The counting data have been expressed as n (%), and the comparison of components was conducted using the χ^2^ test. The correlation of characteristic variables was analyzed using a heatmap. LASSO regression algorithm and 10-fold cross-validation were used to select feature variables. Random Forest, SVM, and XGBoost machine learning were used to rank the importance of feature variables. A Venn diagram was used to identify intersecting features. The diagnostic efficacy of three machine learning algorithms was assessed using receiver operating characteristic (ROC) analysis. Multivariate regression analysis was performed on the intersecting features to construct a nomogram prediction model. The diagnostic performance of the nomogram was evaluated using ROC curves, decision curve analysis, and calibration curves. *P*<0.05 was considered statistically significant.

## 3 RESULTS

### 3.1 Significant Differences in Clinical Characteristics between Benign and Malignant Patients

In this study, 65 patients with TI-RADS class 4 benign thyroid nodules from 60 patients were included, and the proportions of nodules with TI-RADS scores of 4, 5, and 6 were 72.31%, 18.46%, and 9.23%, respectively. There were 20 cases of nodules on the left side and 45 cases on the right side, the proportion of males was 40%, the average age of onset was 48.99±1.87 years, the maximum diameter of nodules was 7.33 mm, the standard deviation was 0.43 mm, and the serum indexes FT3, FT4, FT3/FT4 and TSH were 3.04±0.67 pg/mL, 0.98±0.37 ng/mL, and 4.15±3.15 and 1.45±1.20 μIU/mL, respectively. Among the 52 malignant thyroid nodules detected from 48 patients, 19 were males, and the proportions of nodules with TI-RADS scores of 4, 5, and 6 were 23.08%, 40.39%, and 36.53%, respectively, with 42.31% on the left and 57.69% on the right, and the maximum internal diameter of the nodules was 7.43±0.51 mm; the serum indexes FT3, FT4, FT3/FT4, and TSH were 3.37±2.07 pg/mL, 3.57±1.19 ng/mL, and 1.87±0.89 and 2.21±1.10 μIU/mL, respectively. Compared to the benign nodule patient group, there were statistically significant differences in the serum indicators FT3, FT4, FT3/FT4, TSH, and TI-RADS scores in the malignant nodule patient group (Table **1**).

### 3.2 Comparison of the Multimodal Ultrasound Characteristics and Parameters of Benign and Malignant Thyroid Nodules in Patients with TI-RADS 4 Nodules

Patients with TI-RADS category 4 thyroid malignant nodules exhibit statistically significant differences in their two-dimensional ultrasound image characteristics compared to the benign nodule group, primarily manifested as unclear boundaries (P=0.002), aspect ratio ≥ 1 (*P*<0.001), low internal echoes (P=0.007), and microcalcifications (*P*<0.001). The CEUS imaging characteristics of the malignant nodule group showed statistically significant differences compared to the benign nodule group in terms of enhancement degree (*P*<0.001), circumferential enhancement (P=0.003), and excretion (*P*<0.001). The SWE characteristic parameters Emax (*P*<0.001), Emean (*P*<0.001), and Esd (*P*<0.001) in the malignant nodule group were significantly higher than those in the benign nodule group, and the difference was statistically significant. ∆WiAUC, ∆WiWoAUC, and ∆WoAUC were significantly higher than those in the benign nodules group (*P*<0.001) (Table **2**).

### 3.3 Screening of the Best Matching Factors with LASSO Regression

**Fig. (2):**
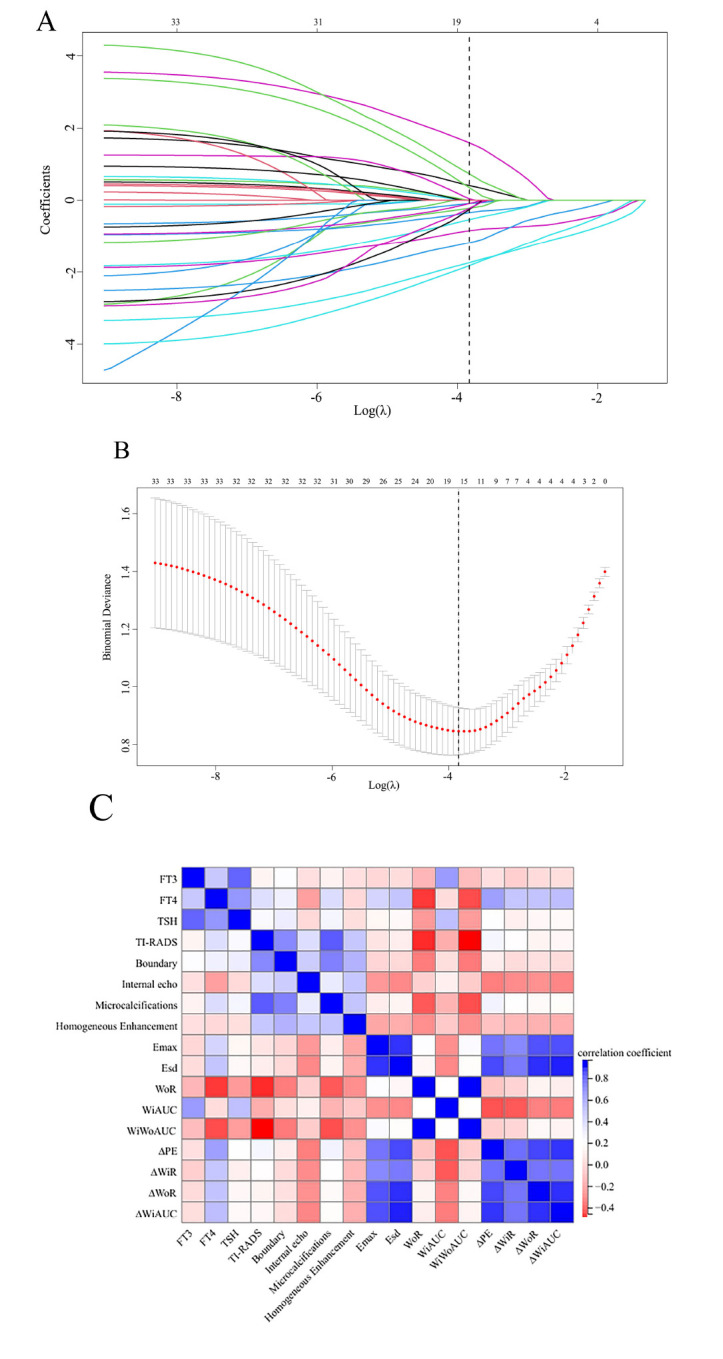
LASSO regression screens the best matching factors. (**A**): Plot of the Lasso regression path; (**B**): 10-fold cross validation; (**C**): Correlation heatmap.

Thirty-six features were screened by 10-fold cross-validation and LASSO regression algorithms, and the LASSO coefficients of each parameter were plotted according to the log(λ) sequence (Fig. **2A**) the vertical lines were plotted on the values selected by 10-fold cross-validation (Fig. **2B**), where the best values were 17 features with non-zero coefficients, namely FT3, FT4, TSH, TI-RADS, internal echo, homogeneous enhancement, boundary, microcalcifications, Emax, Esd, WiAUC, WoR, WiWoAUC, ∆PE, ∆WiR, ∆WoR, and ∆WiAUC. The LASSO dimensionality reduction analysis was conducted on the 17 clinical features and multimodal ultrasound feature parameters were selected, and the results have been presented using a correlation heatmap (Fig. **2C**).

### 3.4 Selection of the Best Predictive Factors using Machine Learning

**Fig. (3):**
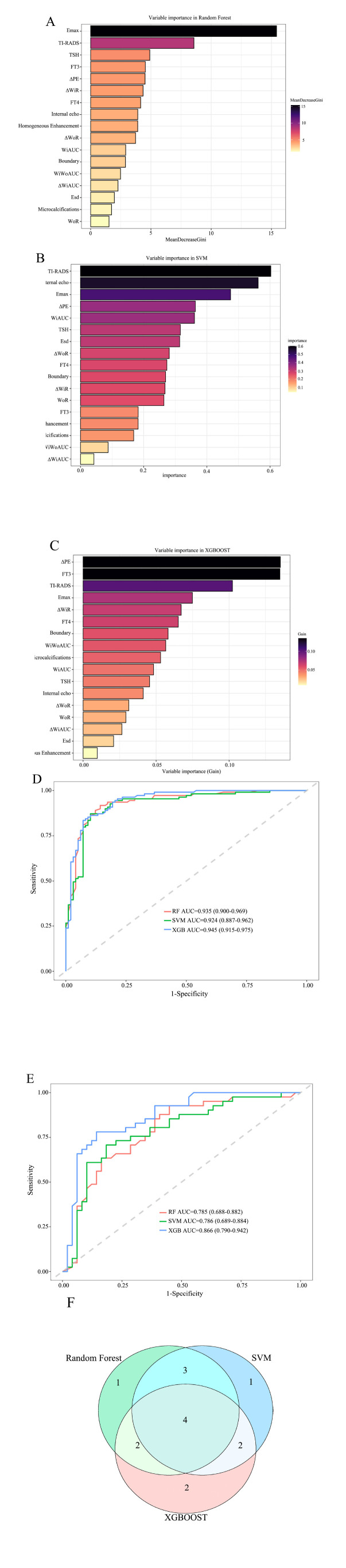
Machine learning selects the best predictive factors. (**A**): Random Forest importance ranking; (**B**): SVM importance ranking; (**C**): XGBOOST importance ranking; (**D**): Training set ROC curve; (**E**): Validation set ROC curve; (**F**): Venn diagram.

We utilized three widely used machine learning methods in bioinformatics, i.e., Random Forest, XGBoost, and SVM, to rank the importance of 17 variables selected by LASSO regression (Fig. **3A**-**C**). The performance of each algorithm was then evaluated on both the training and validation sets using ROC curves (Fig. **3D**, **E**). The results showed that the XGBOOST machine learning algorithm had the best performance in the training set [AUC=0.945 (0.915-0.975)] and the validation set [AUC=0.866 (0.790-0.942)]. The intersection of the top 10 feature parameters ranked by importance for the three machine learning algorithms in the Venn diagram (Fig. **3F**) yielded four optimal predictive factors, including FT4, TI-RADS, Emax, and ∆PE.

### 3.5 Construction and Validation of the Prediction Model

**Fig. (4):**
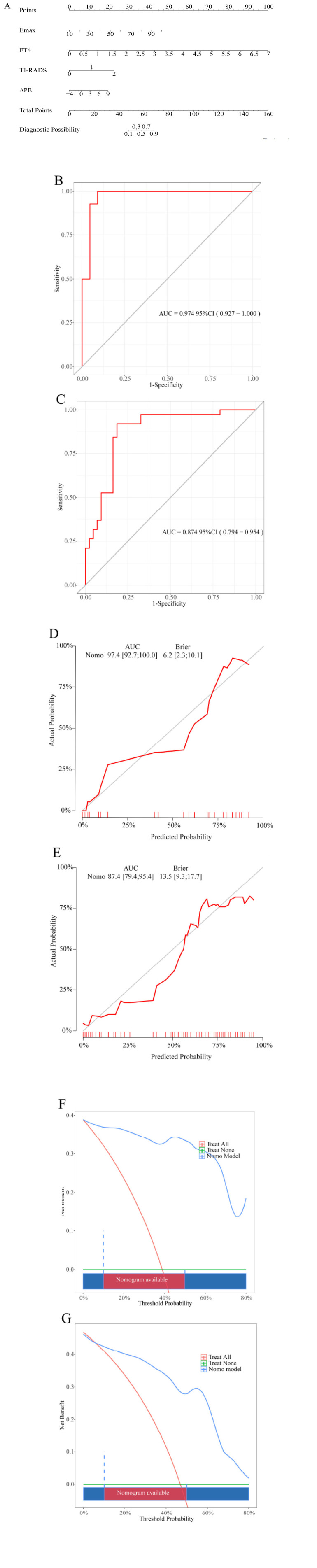
Construction and validation of prediction model. **Note:** **A**: Nomogram prediction model; **B**: Training set ROC curve; **C**: Validation set ROC curve; **D**: Training set calibration curve; **E**: Validation set calibration curve; **F**: Training set DCA curve; **G**: Validation set DCA curve.

The four best predictors selected were analyzed by logistic multiple regression analysis (Table **3**), and the nomogram prediction model (Fig. **4A**) was constructed. In the nomogram, each value of the independent variable corresponded to a score in the top row, and the total score was obtained by summing the scores of the individual independent variables, after which the probability of malignancy for TI-RADS category 4 thyroid nodules was calculated based on the total score. Finally, the ROC curve (Fig. **4B**, **C**), calibration curve (Fig. **4D**, **E**), and DCA curve (Fig. **4F**, **G**) were used to evaluate the prediction performance of nomogram in the training set and validation set. The ROC results showed that the nomogram prediction model had good prediction performance in the training set [AUC=0.974 (0.927-1.000)] and the validation set [AUC=0.874 (0.794-0.954)]. The calibration curve indicated that the calibration curve of the nomogram prediction model closely fit the perfect calibration line, suggesting the model's predictions to align with the actual observations. The results of the DCA decision curve indicated that in the training set, the model exhibited a high positive net benefit when the threshold probability was between 10% and 50%, consistent with the results shown in the validation set.

## 4 DISCUSSION

Thyroid nodules are a common endocrine disorder in clinical practice, with their incidence showing an upward trend in the general population. Currently, fine-needle aspiration (FNA) cytology is a crucial preoperative tool for assessing the nature of thyroid nodules. The Bethesda Reporting System for Thyroid Cytology is the globally recognized standardized evaluation system for FNA results, categorizing cytological diagnoses into six classes, each corresponding to specific ranges of malignant risk, thereby providing critical evidence for clinical decision-making [21]. However, the malignant risk in Bethesda Category III varies significantly across different studies (10%-77.7%), particularly in specialized tumor hospitals where it can reach as high as 77.67% [22]. With the continuous development of high-frequency ultrasound technology, the detection rate of nodules has been increasing year by year. In order to standardize the diagnosis of thyroid nodules, scholars from different countries have successively proposed risk stratification guidelines for thyroid nodules, among which the ACR TI-RADS risk stratification guideline has a lower probability of unnecessary FNA compared to other guidelines [23, 24]. According to the ACR TI-RADS stratification guidelines, the probability of malignant nodules in category 4 is 5%-20%; thus, enhancing the diagnosis of thyroid nodules in this category holds greater clinical significance. Research indicates that ultrasound contrast and elastography can enhance the diagnostic efficacy of thyroid nodules, and their combined application can further improve the diagnostic efficacy of ultrasound contrast and SWE [25, 26]. However, although contrast-enhanced ultrasound and elastography can complement each other in some ways, there is some overlap in the diagnosis of benign and malignant nodules. For example, uneven enhancement or low enhancement shown by ultrasound contrast imaging, along with high stiffness displayed by elastography, may occur simultaneously in certain nodules; thus, they cannot completely differentiate between benign and malignant nodules [27]. In recent years, the integration of ultrasound instruments with machine learning for diagnostic purposes has become a popular trend, with machine learning serving as the “third eye” in medical imaging diagnosis, playing an important role in clinical medical diagnostics. Based on this, this study utilized machine learning algorithms to integrate clinical parameters, two-dimensional conventional ultrasound features, CEUS image features, and SWE image features of patients with TI-RADS category 4 thyroid nodules to construct a clinical prediction model for malignant thyroid nodules of TI-RADS category 4. Incorporating the clinical parameters of patients, two-dimensional conventional ultrasound features, CEUS image features, and SWE image feature parameters into three machine learning methods, including Random Forest, XGBOOST, and SVM, it was found that the XGBOOST learning algorithm exhibited the strongest capability in classifying patients with malignant thyroid nodules compared to other machine learning algorithms. The patient's TI-RADS score, clinical serum indicator FT4, SWE characteristic parameter Emax, and CEUS characteristic parameter ∆PE were found to be the best predictive factors for predicting TI-RADS category 4 thyroid malignant nodules.

Routine ultrasound examinations include two-dimensional grayscale ultrasound and color Doppler ultrasound. Among them, two-dimensional grayscale ultrasound can display the anatomical structure of thyroid tissue based on differences in acoustic impedance, including the size, location, boundaries, shape, capsule, aspect ratio, and internal echo characteristics of thyroid nodules, demonstrating a high diagnostic efficacy for thyroid tissue. The TI-RADS was initially proposed by Horvath *et al*. [28] for the assessment of malignant risk and classification diagnosis of thyroid nodules, categorizing thyroid nodules into the following six types: TI-RADS class 1: normal thyroid; TI-RADS class 2: benign nodules (malignancy rate 0); TI-RADS class 3: probability of benign nodules (malignancy<5%); TI-RADS class 4: suspected malignant nodules [4a (malignancy rate 5%~10%) and 4b (malignancy rate 10%~80%)]; TI-RADS class 5: probability of malignant nodules (malignancy rate > 80%); TI-RADS class 6: malignant nodule confirmed by biopsy. In this study, we found that the higher the TI-RADS score, the greater the probability that the nodule is malignant. Furthermore, FT4, as a major component in the biological effects of thyroid hormones in the body, is primarily related to changes in thyroid function concerning thyroid nodules. When thyroid function is abnormal, such as in hyperthyroidism, it can lead to elevated FT4 levels, which may be associated with the formation and development of thyroid nodules. In this study, the FT4 levels in patients with malignant nodules were significantly higher than those in patients with benign nodules, a result consistent with the findings of Hu *et al*. [29]. The main pathological type of benign thyroid nodules is thyroid adenoma, which is a localized tumor formed by the abnormal hyperplasia of thyroid follicular epithelium; therefore, benign nodules often do not cause abnormal changes in thyroid hormones. The pathological types of malignant thyroid nodules are primarily papillary carcinoma, follicular carcinoma, undifferentiated carcinoma, and medullary carcinoma, which typically lead to abnormal changes in thyroid hormones [30].

SWE, a non-invasive method for assessing tissue stiffness, generates transverse shear waves within the tissue and measures the shear wave velocity, which is then converted into Young's modulus. The obtained Young's modulus can directly assess the stiffness of nodules, visually displaying the stiffness of thyroid nodules in relation to the surrounding thyroid tissue. It offers various advantages, such as real-time assessment, efficiency, and reproducibility, and is currently widely used in the diagnosis of various clinical diseases [31]. When thyroid nodules undergo malignant transformation, their histological characteristics change, leading to decreased elasticity and increased hardness. Literature indicates that the hardness of the nodules is positively correlated with their degree of malignancy; the greater the maximum value of the Young's modulus of the nodule, the higher the risk of malignancy [32]. Different studies have also shown discrepancies regarding the threshold for thyroid nodules [32, 33]. Therefore, there has yet to be a guideline providing the standard diagnostic threshold for the Young's modulus in the diagnosis of thyroid nodules. By incorporating the SWE quantitative parameters of this study into three machine learning algorithms, it was found that Emax ranked high in importance among the three algorithms, demonstrating its great potential in diagnosing TI-RADS class 4 malignant thyroid nodules. This result has been found to be similar to the findings of Zhang *et al*. [33]. However, the Emax value in this study was significantly higher than the results of Petersen M *et al*. [34], which may be due to the fact that the nodules included in this study were mainly solid nodules. When thyroid tissue lesions occur, the follicular epithelium undergoes fibrosis and calcification, tissue elasticity decreases, and tissue hardness increases.

CEUS is based on real-time observation of the blood flow inside and around nodules to determine their benign or malignant nature [35]. Its image analysis includes the following aspects: (1) uniformity of enhancement, categorized as uniform enhancement or non-uniform enhancement (including areas of local non-enhancement), with particular attention to the uniformity of internal enhancement at the peak enhancement of the lesion; (2) degree of enhancement, classified as high, equal, or low enhancement, focusing on the comparison of the lesion’s echo intensity with that of the surrounding thyroid parenchyma at the point of maximal enhancement. Malignant thyroid nodules, due to the rapid formation of new blood vessels, have disordered and irregular structures, resulting in uneven enhancement in most cases. If microcalcifications and interstitial fibrosis are present, they may exhibit low or uneven enhancement [35]. Vue Box, as an external offline perfusion analysis software, integrates calibration to evaluate the wash-in and wash-out dynamics of microvessels in more detail. Time intensity curve (TIC) parameters are calculated through perfusion analysis [36]. The CEUS image features selected in this study did not obtain effective factors for predicting model construction after machine learning ensemble analysis. Instead, the TIC parameter ∆PE performed the most outstandingly.

In this study, we integrated multiple ultrasound feature parameters of TI-RADS 4 patients to meaningfully reflect their malignant nodule characteristics. Through screening, all indicators were included in the model construction. On the basis of selecting the optimal predictor variables, constructing a logistic regression model and a column chart can effectively predict the likelihood of the occurrence of TI-RADS 4 types of malignant thyroid nodules.

## 5 STUDY LIMITATION

This study has involved certain limitations: first, it was a single-center retrospective study, which may have introduced selection bias and limited the generalizability of the results. Second, since TI-RADS 4-category nodules are classified as “moderately suspicious for malignancy”, their prevalence in the thyroid nodule population is limited, and their malignant pathological types are predominantly papillary thyroid carcinomas. Consequently, the number of TI-RADS 4-category nodules included in this study was limited, and all malignant nodules were papillary thyroid carcinomas. Therefore, this study model may be more applicable for the preoperative diagnosis of papillary thyroid carcinoma in TI-RADS 4-category nodules. In subsequent studies, we will collaborate with 2-3 medical institutions of different levels to conduct a multicenter study and extend the study duration to increase the sample size. By utilizing multicenter data to cover a more comprehensive range of pathological types, we aim to validate the model's diagnostic stability for different malignant tumors. Additionally, by collecting independent external datasets, we will focus on verifying the model's diagnostic consistency across heterogeneous data. Second, we will simultaneously incorporate more comprehensive clinical information (such as patient age, laboratory test results, etc.) in external validation to further optimize the model's adaptability.

## CONCLUSION

In this study, we have developed a column chart prediction model to assess the risk of deterioration in patients with TI-RADS class 4 thyroid nodules. The research results can provide a reference for clinicians to detect the risk of nodule deterioration on the basis of clinical features, two-dimensional ultrasound features, and CEUS and SWE features, and achieve timely intervention for high-risk patients. It can serve as a convenient, practical, and effective clinical decision-making tool.

## Figures and Tables

**Fig. (1):**
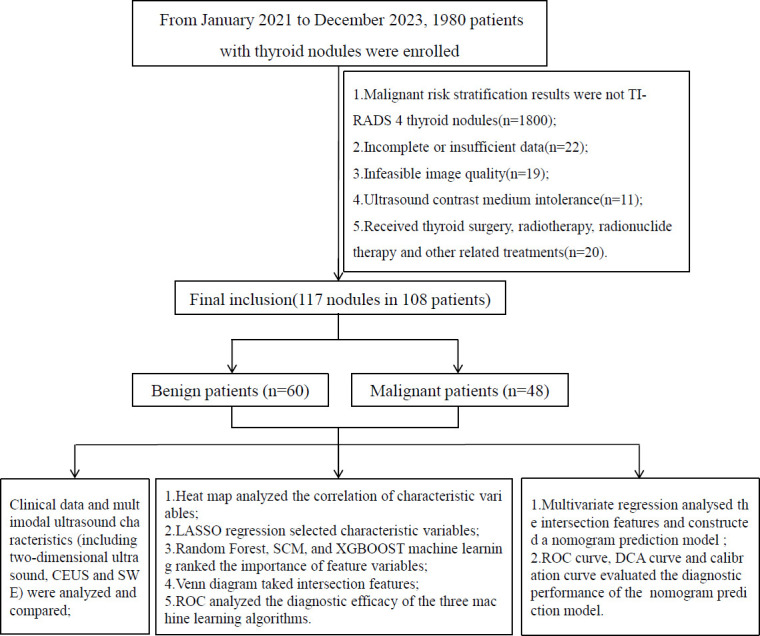
Flowchart of subject enrolment and study design.

**Table 1:** Comparison of clinical characteristics between TI-RADS 4 thyroid nodules benign and malignant patients.

**Variables**		**Benign Patient** **(n=60)**	**Malignant Patient** **(n=48)**	**t/χ2**	***P***
Age		48.99±1.87	49.57±1.79	-1.699	0.092
Sex	Male	24(40.00)	19(39.58)	0.002	0.965
	Female	36(60.00)	29(60.42)		
Location	Left	20(30.77)	22(42.31)	1.671	0.196
	Right	45(69.23)	30(57.69)		
Maximum diameter		7.33±0.43	7.43±0.51	-1.151	0.252
FT3 (pg/mL)		3.04±0.67	3.37±2.07	-1.210	0.229
FT4 (ng/mL)		0.98±0.37	3.57±1.19	-15.236	<0.001
FT3/FT4		4.15±3.15	1.87±0.89	6.775	<0.001
TSH (μIU/mL)		1.45±1.20	2.21±1.10	-3.580	<0.001
TI-RADS	4	47(72.31)	12(23.08)	28.889	<0.001
	5	12(18.46)	21(40.39)		
	6	6(9.23)	19(36.53)		

**Table 2:** Comparison of the multimodal ultrasound characteristics and parameters of benign and malignant thyroid nodules in patients with TI-RADS 4.

**Variable**			**Benign Nodule** **(n=65)**	**Malignant Nodule** **(n=52)**	**t/Z/χ^2^**	***P***
Two-dimensional ultrasonic features	Boundary	Clear	41(63.08)	18(34.62)	9.361	0.002
		Unclear	24(36.92)	34(65.38)		
	Aspect ratio	<1	44(67.69)	12(23.08)	23.044	<0.001
		≥1	21(32.31)	40(76.92)		
	Internal echo	low	28(43.08)	37(71.15)	12.254	0.007
		Equal	6(9.23)	4(7.69)		
		Heigh	15(23.08)	2(3.85)		
		Mixed	16(24.61)	9(17.31)		
	Microcalcifications	Yes	20(30.77)	41(78.85)	26.758	<0.001
		No	45(69.23)	11(21.15)		
CEUS features	Enhancement degree	High	14(21.54)	12(23.08)	13.347	<0.001
		low	20(30.77)	31(59.62)		
		Equal	31(47.69)	9(17.30)		
	Homogeneous enhancement	Yes	21(32.31)	14(26.92)	0.400	0.527
		No	44(67.69)	38(73.08)		
	Pattern of enhancement	Centripetal	16(24.62)	13(25.00)	0.298	0.862
		Eccentricity	9(13.85)	9(17.31)		
		Diffuse	40(61.53)	30(57.69)		
	Circumferential enhancement	Yes	15(23.08)	2(3.85)	8.603	0.003
		No	50(76.92)	50(96.15)		
	Excretion	Fastly	10(16.92)	39(75.00)	40.450	<0.001
		Slowly	37(56.92)	7(13.46)		
		Synchronously	18(26.16)	6(11.54)		
SWE parameters	Emax		31.20(28.32,34.26)	49.01(38.74,56.88)	-5.894	<0.001
	Emean		22.40(19.44,25.61)	33.06(25.63,39.29)	-5.033	<0.001
	Emin		15.62(11.07,20.32)	16.095(9.89,22.68)	-0.014	0.989
	Esd		3.930(2.750,5.140)	8.890(6.342,10.985)	-6.906	<0.001
CEUS parameters	PE		37.25±3.71	34.07±3.41	4.775	<0.001
	WiR		34.40(32.35,36.27)	28.52(26.31,30.58)	-7.276	<0.001
	WoR		28.99(28.29,30.25)	25.95(23.86,27.86)	-5.806	<0.001
	WiAUC		45.39±5.22	40.88±3.15	5.775	<0.001
	WiWoAUC		47.03(45.46,48.65)	43.96(42.18,45.58)	-5.612	<0.001
	WoAUC		47.95±4.29	46.81±4.18	1.445	0.151
	WiPI		36.71±3.98	33.97±4.08	3.660	<0.001
	∆PE		-0.80(-1.27,-0.29)	3.18(1.37,4.58)	-9.147	<0.001
	∆WiR		0.16(-1.20,1.02)	3.16(1.41,4.84)	-7.509	<0.001
	∆WoR		0.28(-1.76,1.15)	3.05(2.47,3.89)	-7.106	<0.001
	∆WiAUC		-0.02(-1.17,0.91)	3.18(1.74,4.91)	-7.482	<0.001
	∆WiWoAUC		0.23(-0.78,0.79)	3.49(2.10,5.07)	-8.061	<0.001
	∆WoAUC		0.56(-1.68,1.51)	3.43(2.26,4.22)	-6.928	<0.001
	∆WiPI		-0.31(-1.14,0.30)	3.46(2.09,5.22)	-9.075	<0.001

**Note:** Emax: Maximum Young's modulus; Emean: Average Young's modulus; Emin: Minimum Young's modulus; Esd: Elastic Standard Deviation; PE: Peak intensity; WiR: Washing input ratio; WoR: Washing-out ratio; WiAUC: The area under the wash-in curve; WiWoAUC: The area under the wash-in and wash-out curve; WoAUC: The area under the washout curve; WiPI: Influx perfusion index; ∆PE: Difference in PE between normal glands and nodules; ∆WiR: WiR difference between normal glands and nodules; ∆WoR: WoR difference between normal glands and nodules; ∆WiAUC: WiAUC difference between normal glands and nodules; ∆WiWoAUC: WiWoAUC difference between normal glands and nodules; ∆WoAUC: WoAUC difference between normal glands and nodules; ∆WiPI: WiPI difference between normal glands and nodules.

**Table 3:** Multivariate regression analysis.

**Variable**	**β**	**SE**	**Wald χ^2^**	**OR**	**95%CI**	***P***
Emax	0.211	0.102	4.239	1.234	1.010-1.507	0.04
FT4	3.018	1.175	6.597	20.450	2.044-204.588	0.01
TI-RADS	2.421	1.151	4.424	1.645	1.179-2.411	0.035
∆PE	0.498	0.195	6.522	1.645	1.123-2.411	0.011

**Note:***β*: Regression coefficient;* SE*: Standard Error; OR: Odds Ratio; CI: Confidence Interval.

## Data Availability

All data generated or analyzed during this study are included in this published article.

## LIST OF ABBREVIATIONS

TI-RADS: Thyroid Imaging Reporting and Data System
SWE: Shear wave elastography
CEUS: Contrast-enhanced ultrasound
SVM: Support vector machine
XGBoost: eXtreme gradient boosting
LASSO: Least absolute shrinkage and selection operator
FT4: Free thyroxine 4
PE: Peak enhancement

## STANDARDS OF REPORTING

STROBE guidelines were followed.
